# Rock-salt-type lithium metal sulphides as novel positive-electrode materials

**DOI:** 10.1038/srep04883

**Published:** 2014-05-08

**Authors:** Atsushi Sakuda, Tomonari Takeuchi, Kazuhiro Okamura, Hironori Kobayashi, Hikari Sakaebe, Kuniaki Tatsumi, Zempachi Ogumi

**Affiliations:** 1 Research Institute for Ubiquitous Energy Devices, National Institute of Advanced Industrial Science and Technology (AIST), 1-8-31 Midorigaoka, Ikeda, Osaka 563-8577, Japan; 2 Office of Society-Academia Collaboration for Innovation, Kyoto University, Gokasho, Uji, Kyoto 611-0011, Japan

## Abstract

One way of increasing the energy density of lithium-ion batteries is to use electrode materials that exhibit high capacities owing to multielectron processes. Here, we report two novel materials, Li_2_TiS_3_ and Li_3_NbS_4_, which were mechanochemically synthesised at room temperature. When used as positive-electrode materials, Li_2_TiS_3_ and Li_3_NbS_4_ charged and discharged with high capacities of 425 mA h g^−1^ and 386 mA h g^−1^, respectively. These capacities correspond to those resulting from 2.5- and 3.5-electron processes. The average discharge voltage was approximately 2.2 V. It should be possible to prepare a number of high-capacity materials on the basis of the concept used to prepare Li_2_TiS_3_ and Li_3_NbS_4_.

Lithium-ion batteries with high energy densities are highly desirable as power sources for electric vehicles and electric power storage devices^1^. The development of new high-capacity active materials for electrodes is essential for fabricating such batteries.

Positive electrodes, such as those made of layered and spinel-type lithium metal oxides and lithium metal phosphates, have been used in lithium-ion batteries. These electrodes usually have one equivalent of lithium per transition metal or less, and their capacities are less than 200 mA h g^−1^. Of late, layered lithium metal oxides with more than one equivalent of lithium per transition metal are being actively researched and developed as novel positive-electrode materials with capacities greater than 200 mA h g^−1^. However, it is still difficult to achieve reversible capacities greater than 300 mA h g^−1^ in layered lithium metal oxides. Transition metals act as the redox centre in many conventional active materials. In most cases, a single-electron reaction occurs during charging and discharging, and multielectron reactions, which are necessary for higher capacities, do not take place. This is partly because of (i) the instability of the active materials after the multielectron reactions, particularly in the charged state, where the higher valence state of transition metals is contained, and (ii) the irreversibility of the structures against larger changes in the valence state of the constituent metal ions. However, several exceptions have been reported. For instance, it had been reported that lithium vanadium oxides, ω-Li_x_V_2_O_5_, show a capacity of more than 300 mA h g^−1^ and that approximately 3 lithiums per formula unit can be insert and removed from them; this corresponds to 1.5 electron processes per vanadium atom^2^. Although most positive-electrode materials charge and discharge mainly through the redox reaction of the transition metal, the redox reaction of anions as well as that of the transition metals should be exploited to achieve higher capacities. That is to say, it is essential that charge and discharge reactions involving more than two electrons should occur in the electrode materials. Therefore, the challenge is to develop new active materials that can charge and discharge through processes that involve more than two electrons.

Conversion electrodes, which involve the formation and decomposition of Li_2_O, Li_2_S, or LiF and the reduction and oxidation of metal nanoparticles, exhibit high capacities because their constituent materials undergo multielectron reactions^3^,^4^,^5^. Most conversion electrodes show low potential and thus are usually employed as negative electrodes. A few conversion electrode materials, such as metal fluorides, show high capacities and voltages^5^. However, their reversibility is usually low because of the large structural changes that occur during charging and discharging.

Metal sulphides have also been studied as potential positive-electrode materials^6^,^7^,^8^,^9^. An advantage of metal sulphides is that their capacity is related to the sulphur redox reaction in addition to the redox reaction of the transition metal; this leads to a high capacity owing to the multielectron redox reaction. For example, crystalline TiS_3_ charges and discharges with reversible capacities greater than 300 mA h g^−1^, a value that corresponds to processes involving more than two electrons^6^,^7^,^8^,^9^. However, it has been reported that the reversibility of TiS_3_ is low, probably owing to the transformation of its polyhedral structure from a trigonal prism to a more stable octahedron^6^,^7^,^8^. It was recently reported that the amorphisation of TiS_3_ via mechanical milling improves its performance as an electrode material^10^,^11^,^12^. Amorphous TiS_3_ exhibits a three-dimensional framework, which would be favourable for stabilising its structure against the large volume changes caused by the charge-discharge process. The development of electrode materials with structures that remain stable during multielectron processes is thus strongly desired.

We focused on the lithium predoping of metal sulphide electrodes because (i) it might stabilise the structure and (ii)a lithium-containing positive-electrode material is highly desirable; commercial negative electrodes usually do not contain lithium. In contrast to lithium metal oxides and metal sulphides, lithium-containing metal sulphides have not been widely studied; a few exceptions are Li_2_FeS_2_ and LiTi_y_M_1−y_S_2_ (M = V, Cr, or Fe), etc.^13^,^14^,^15^. The development of new lithium-containing metal sulphides (Li_x_MS_y_) has been a challenge. However, it should be possible to fabricate new crystalline-phase materials with high capacities and good structural reversibility by extracting/inserting Li in lithium-containing metal sulphides.

Here, we report the development of two novel lithium transition metal sulphides, Li_2_TiS_3_ and Li_3_NbS_4_, which were fabricated *via* mechanochemical synthesis. The structures and electrode performances of the sulphides were investigated. We found that Li_2_TiS_3_ and Li_3_NbS_4_ exhibited reversible charging and discharging with high capacities of 425 and 386 mA h g^−1^, respectively; these values correspond to processes involving 2.5 and 3.5 electrons, respectively.

## Results

**Figure 1:**
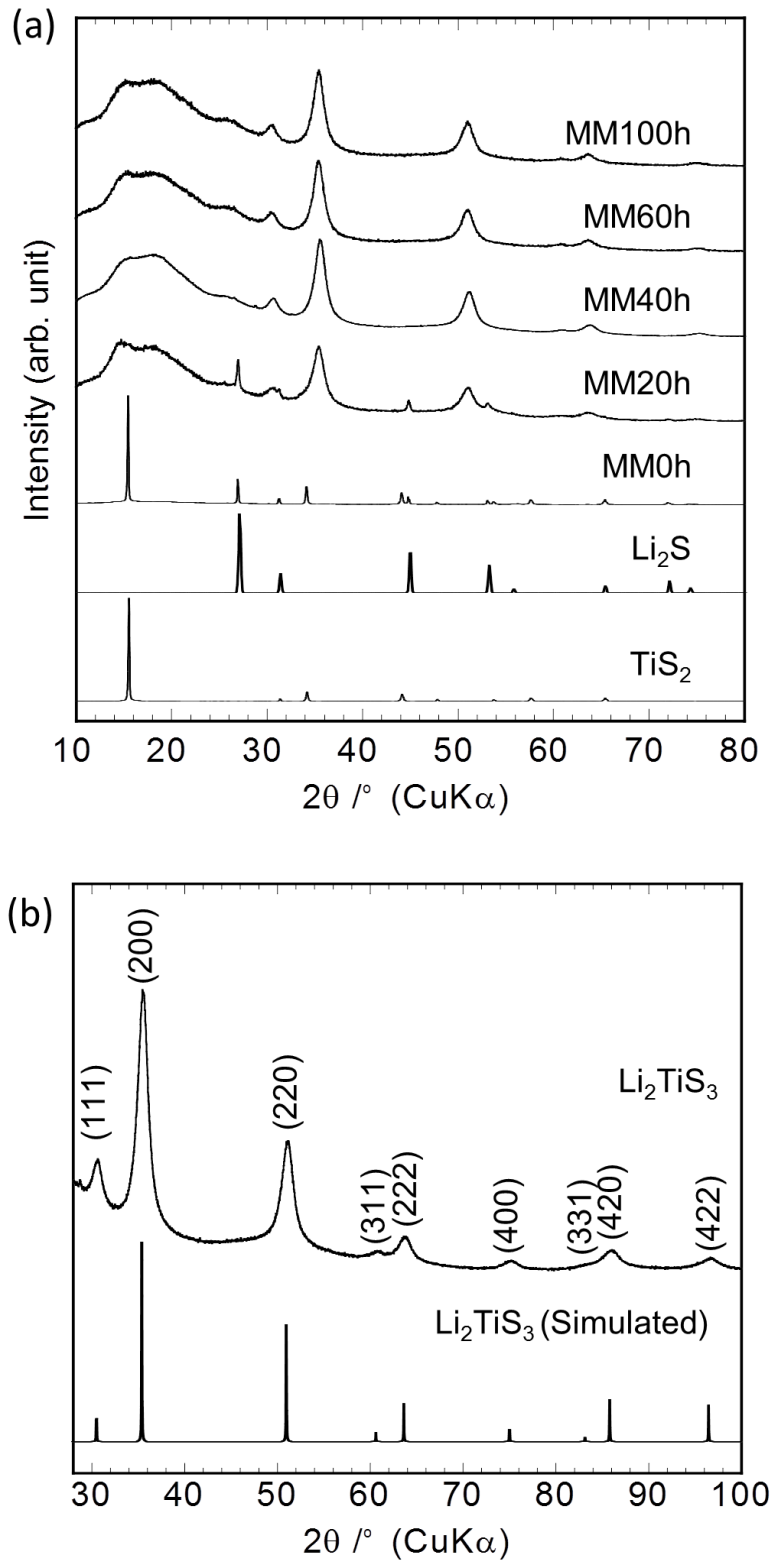
XRD patterns of Li_2_TiS_3_. (a) XRD patterns of TiS_2_, Li_2_S, a mixture of TiS_2_ and Li_2_S that was not mechanically milled (0 h), and Li_2_TiS_3_ samples prepared by mechanical milling (MM) for 20, 40, 60, and 100 h. (b) XRD pattern of Li_2_TiS_3_ prepared by MM for 40 h and the simulated XRD pattern of Li_2_TiS_3_ with a rock-salt-type structure (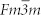).

Figure 1a shows the X-Ray diffraction (XRD) patterns of TiS_2_, Li_2_S, a mixture of TiS_2_ and Li_2_S that was not mechanically milled (0 h), and Li_2_TiS_3_ samples prepared by mechanical milling (MM) for 20, 40, 60, 80, and 100 h. Diffraction peaks corresponding to TiS_2_ and Li_2_S were not evident after the milling process; however, several new peaks appeared. The XRD patterns of Li_2_TiS_3_ after MM for 40, 60, and 100 h were similar. The broad peak observed at 2*θ* = 10–25° was owing to the Kapton® film.

Figure 1b shows the XRD pattern of Li_2_TiS_3_ prepared by MM for 40 h and the simulated pattern of Li_2_TiS_3_ with a rock-salt-like structure (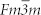). It was assumed that its unit cell contains four formula units of [Li_2/3_/Ti_1/3_]_4a_S_4b_, and the cell parameter is *a* = 5.06 Å. Table 1 shows the simulated powder X-ray data obtained using the software program Powder Cell^16^. The peak positions of the prepared Li_2_TiS_3_ sample were consistent with those of the simulated rock-salt-type Li_2_TiS_3_. This suggests that the synthesised Li_2_TiS_3_ sample had a rock-salt-type structure. Furthermore, the intensity ratios of the XRD peaks of the prepared Li_2_TiS_3_ sample were in good agreement with the simulated ones for Li_2_TiS_3_, indicating that the Ti occupancy in the 4a sites was approximately 0.33. Pattern fitting, performed using the program RIETAN-2000^17^, also indicated that the Li and Ti occupancies in the 4a sites were approximately 0.665(4) and 0.335(4), respectively. To the best of our knowledge, this is the first report of a lithium titanium sulphide with a rock-salt-like structure.

**Figure 2:**
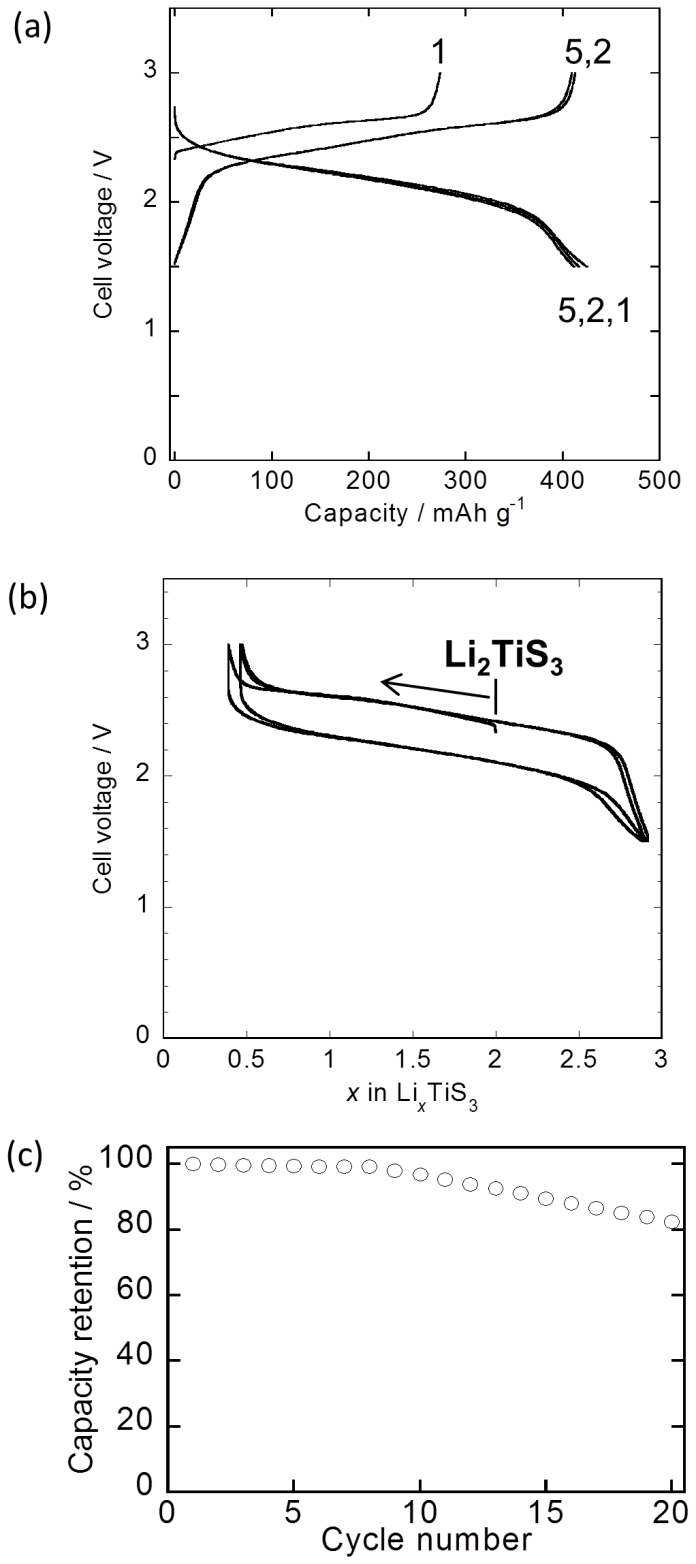
Electrode performance of Li_2_TiS_3_. (a) Charge-discharge curves of the cell fabricated using Li_2_TiS_3_. Cut-off voltages were 1.5–3.0 V (versus a Li counter electrode). (b) Charge-discharge curves, with the number of lithium atoms per formula unit as the X-axis. (c) Cycling performance of the cell fabricated using Li_2_TiS_3_.

The rock-salt-type Li_2_TiS_3_ was employed as an electrode active material for lithium secondary batteries. Figure 2a shows the charge-discharge curves for the first 5 cycles of the cells fabricated using Li_2_TiS_3_. The initial charge and discharge capacities were 273 and 425 mA h g^−1^, respectively. Figure 2b shows the charge-discharge characteristics as a function of the lithium content. It was found that approximately 1.6 lithium atoms per formula unit could be extracted during the initial charging process and 2.5 lithium atoms could be inserted into the structure during the initial discharging process. The extraction and insertion of 2.5 lithium atoms into the structure of Li_2_TiS_3_ was reversible during repeated cycling. Thus, this electrode active material could contain more than 2 lithium atoms in its structure; the structure could be charged and discharged such that it ranged from Li_0.4_TiS_3_ to Li_2.9_TiS_3_ over voltages of 1.5–3.0 V. The initial charging and discharging curve exhibited significant overlap with the second and third curves for the structure ranging from Li_0.4_TiS_3_ to Li_2_TiS_3_, suggesting that the charge-discharge mechanism remained unchanged. Figure 2c shows the cycle performance of the cell fabricated using Li_2_TiS_3_. The discharge profiles did not change drastically over 5 cycles; however, the capacity of the cell faded after the 10^th^ cycle.

**Figure 3:**
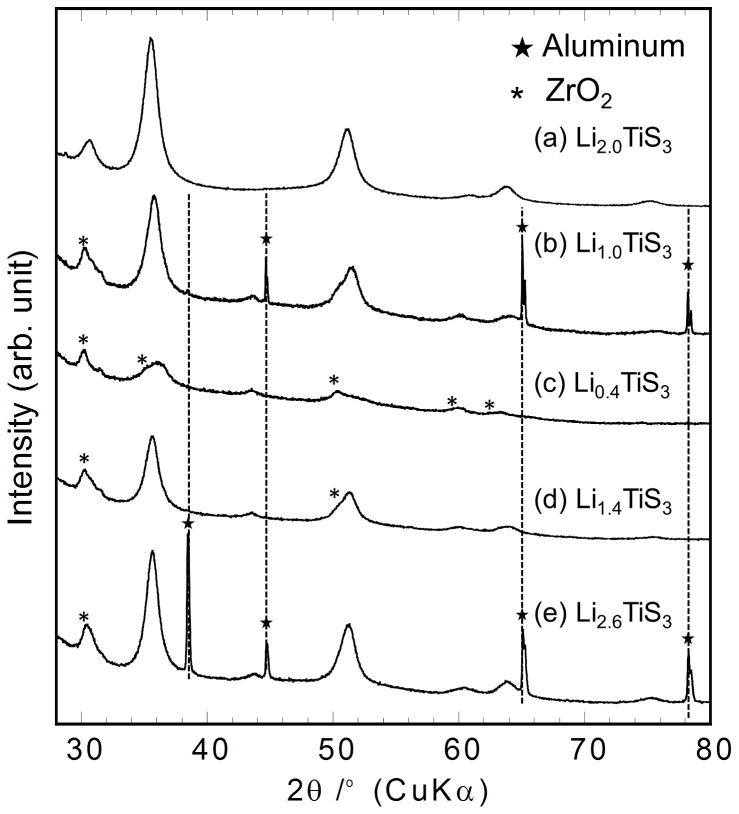
XRD patterns of Li*_x_*TiS_3_ before and after the charge-discharge measurements. The patterns correspond to *x* = (a) 2.0, (b) 1.0, (c) 0.4, (d) 1.4, and (e) 2.6.

To further investigate the electrochemical reactions occurring during the charge-discharge processes, *ex situ* XRD measurements were performed for Li*_x_*TiS_3_ with *x* = 2.0, 1.0, 0.4, 1.4, and 2.6 (Figure 3). The peaks became broad after a 1.0-electron charge, as shown in Figure 3b. Further, the peaks became even broader and decreased in intensity after the charge to Li_0.4_TiS_3_ (Figure 3c). Some of these broader, lower-intensity peaks in Figure 3c were identified as being attributable to ZrO_2_, which could have contaminated the test samples during the ball-milling process. Finally, after the discharge to Li_1.4_TiS_3_ (Figure 3d) and Li_2.6_TiS_3_ (Figure 3e), the peaks became sharper and increased in intensity. These results indicate that the extraction of lithium atoms from Li_2_TiS_3_ results in amorphisation and that reverse reactions occur during the discharge process. It should be noted that the XRD pattern corresponding to the rock-salt-type sample was observed after discharging to Li_2.6_TiS_3_. This suggests that the volume change during charge and discharge can be expected to occur in a three-dimensional manner.

**Figure 4:**
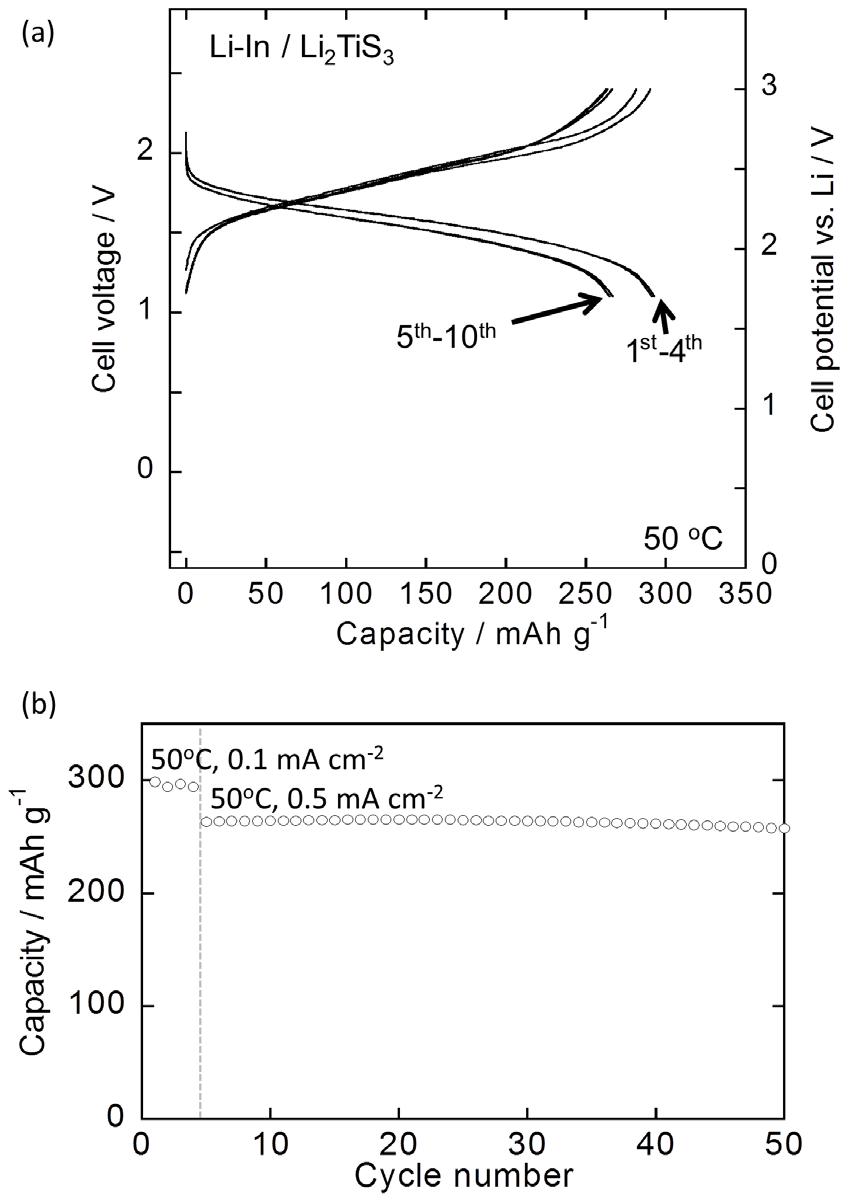
Electrode performance of Li_2_TiS_3_ in an all-solid-state cell. (a) Charge-discharge curves of the all-solid-state cell fabricated using Li_2_TiS_3_ at 50°C. Cut-off voltages were 0.9–2.4 V (versus a Li-In counter electrode). The cell was precycled at 30°C. Current densities were 0.1 mA cm^−2^ for the 1^st^–4^th^ cycles and 0.5 mA cm^−2^ after the 5^th^ cycle. (b) Cycling performance of the all-solid-state cell fabricated using Li_2_TiS_3_ at 50°C.

To evaluate the cyclability of Li_2_TiS_3_, an all-solid-state cell was constructed using Li_2_TiS_3_ as the electrode material. It is known that a number of electrode materials show high cyclability when used in all-solid-state cells. Figure 4a shows the charge-discharge curves of the all-solid-state cell at 50°C. Figure 4b shows the cycling performance of the all-solid-state cell. The cell exhibited high capacity retention, retaining 97% of its capacity from the 5^th^ to the 50^th^ cycle. Thus, Li_2_TiS_3_ exhibits high reversibility when subjected to charge-discharge cycling.

**Figure 5:**
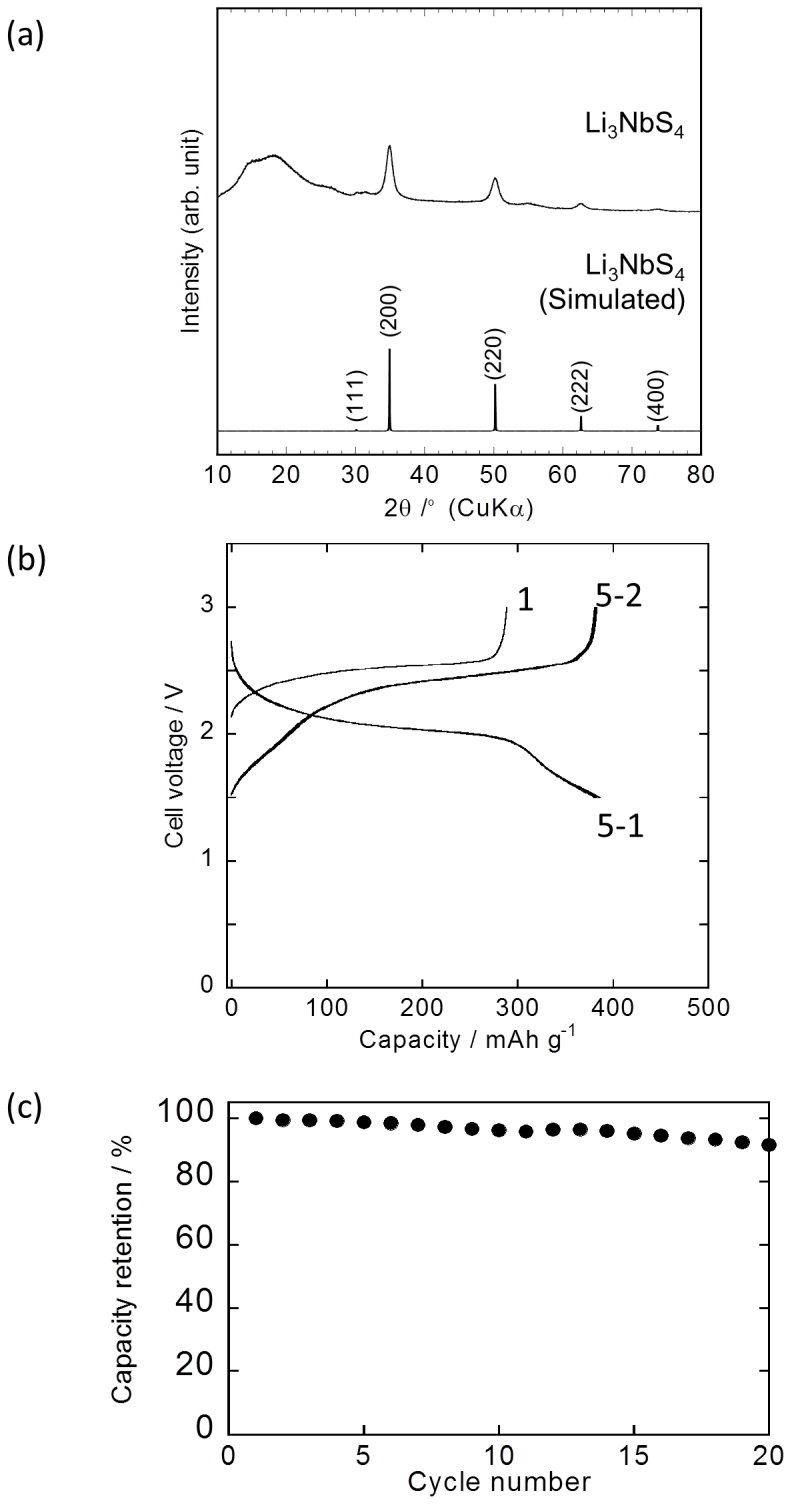
Characterisation of Li_3_NbS_4_. (a) XRD pattern of Li_3_NbS_4_ prepared by MM for 40 h and the simulated XRD pattern of Li_3_NbS_4_ with a rock-salt-type structure (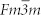) obtained using the cell parameter *a* = 5.13. (b) Charge-discharge curves of the cell fabricated using Li_3_NbS_4_ and a liquid-based electrolyte. Cut-off voltages were 1.5–3.0 V (versus a Li counter electrode). (c) Cycling performance of the cell fabricated using Li_3_NbS_4_.

A number of novel lithium metal sulphides can be developed through mechanochemical synthesis on the basis of the concept employed for altering the structure of Li_2_TiS_3_. Here, we show an example of one such material, Li_3_NbS_4_, which has a structure similar to that of Li_2_TiS_3_ and was prepared by mechanochemical synthesis from Li_2_S, NbS_2_, and S_8_. Figure 5a shows the XRD pattern of Li_3_NbS_4_. The XRD pattern is similar to that of Li_2_TiS_3_. Therefore, Li_3_NbS_4_ should also have a cubic structure, with the cell parameter *a* = 5.13 Å. Figure 5b shows the charge-discharge curves of the cell fabricated using Li_3_NbS_4_; 1 M LiPF_6_ in a mixture of ethylene carbonate (EC) and dimethyl carbonate (DMC) was used as the electrolyte. The initial charge-discharge behaviour was similar to that of Li_2_TiS_3_. The reversible capacity was 386 mA h g^−1^, which corresponds to 3.5-electron processes. Figure 5c shows the cycling performance of the Li_3_NbS_4_ cell. The cell exhibited better cycling performance than that of the Li_2_TiS_3_ cell; this was despite the fact that the charging and discharging occurred owing to 3.5-electron processes in the case of the former. The electronic conductivities of compressed pellets of powdered Li_2_TiS_3_ and Li_3_NbS_4_ were 8 × 10^−6^ and 2 × 10^−3^ S cm^−1^, respectively. These values are likely related to the electronic conductivities of the compounds.

The elucidation of the charge-discharge mechanism should result in the development of new electrode materials that exhibit better performances. To be able to determine where the excess lithium atoms exist in the sulphides is the most important goal as it will lead to the synthesis of the novel high-capacity electrode materials. Further studies on this topic are currently underway.

The calculated gravimetric energy densities of Li/Li_x_TiS_3_ (0.4 ≤ x ≤ 2.9) and Li/Li_3_NbS_4_ (0.4 ≤ x ≤ 3.9) were 850 and 780 W h kg^−1^. These values are larger than that for a typical positive electrode of LiCoO_2_, for which the energy density of Li/Li_x_CoO_2_ (0.4 ≤ x ≤ 1) is 660 W h kg^−1^. The combination of positive and negative electrodes is an important aspect. In order to use Li_2_TiS_3_ and Li_3_NbS_4_ as positive electrodes, their initial lithium content should be increased, that is, discharged state materials should be prepared, or lithium-containing negative electrodes such as lithium and lithium-alloy should be used. To conclude, in this study, we prepared the novel materials Li_2_TiS_3_ and Li_3_NbS_4_ from Li_2_S, TiS_2_, NbS_2_ and S_8_ via mechanochemical synthesis using ball milling. Li_2_TiS_3_ was used as an electrode active material for a lithium secondary battery; it charged and discharged with initial charge and discharge capacities of 273 and 425 mA h g^−1^, respectively. The average discharge voltage was approximately 2.2 V. Similarly, Li_3_NbS_4_ was also used as an electrode active material and showed a high capacity of 386 mA h g^−1^. Reversible charging and discharging were confirmed, despite the processes being multielectron ones.

We have synthesised only two novel lithium metal sulphides through a mechanochemical method. However, it should be possible to fabricate a number of novel lithium metal sulphides using this method.

## Methods

A mechanochemical synthesis method was employed to fabricate the lithium titanium sulphides. Mechanochemical synthesis involves the promotion of chemical reactions using mechanical energy. There are many merits of this method, such as the ability to prepare nanoparticles directly using a simple homogeneous reaction at room temperature. The primary advantage of mechanochemical synthesis is the ability to obtain the final product in a metastable state, which is difficult through conventional methods. In particular, amorphous materials and materials with highly symmetrical three-dimensional crystal structures can be obtained.

Li_2_TiS_3_ was mechanochemically synthesised at room temperature using a planetary ball mill apparatus (P-7, Fritsch GmbH). Lithium sulphide (Li_2_S, 99.9%, Mitsuwa Pure Chemicals) and titanium disulphide (TiS_2_, 99.8%, Wako Pure Chemical Industries) were used as the starting materials. Appropriate amounts of Li_2_S and TiS_2_ were weighed and mixed, and the mixture (1.5 g) was placed into a zirconia pot (45 mL) along with 500 zirconia balls (4 mm in diameter). The pot was then placed in an argon-filled glove box. The rotation speed of ball milling was fixed at 510 rpm.

The XRD patterns of the synthesised samples were recorded using an X-ray diffractometer (Rotaflex RU-200B/RINT, Rigaku). Prior to the measurements, the samples were covered with Kapton® film in an argon-filled glove box to prevent exposure to air. The electrochemical cells used to test the samples were also constructed in an argon-filled glove box. The working electrodes were prepared from Li_2_TiS_3_ (10 mg), acetylene black (2 mg), and polytetrafluoroethylene (PTFE) powder (0.7 mg). A 1 M solution of LiPF_6_ in a 50:50 (by volume) mixture of EC and DMC (Tomiyama Pure Chemical Industries Ltd.) was used as the electrolyte. The counter electrode consisted of a Li foil disk (15 mm diameter, 0.2 mm thickness). The electrochemical measurements were performed at 30°C using a charge-discharge unit (TOSCAT-3100, Toyo System) at a current density of 10 mA g^−1^ between 1.5 and 3.0 V. The all-solid-state cells were constructed as follows. Li_2_TiS_3_, the glass electrolyte 70(0.75Li_2_S·0.25P_2_S_5_)·30LiI, and acetylene black (AB) were mixed in a weight ratio of 60:30:10 in an agate mortar, and the mixture was used to prepare the positive-electrode material. The glass electrolyte Li_2_S-P_2_S_5_-LiI, which has an ionic conductivity of more than 10^−3^ S cm^−1^^18^,^19^, was used as the solid electrolyte. A lithium-indium alloy was used to make the counter electrode. Bilayered pellets consisting of the positive-electrode material (10 mg) and the glass electrolyte (70 mg) were pressed under a pressure of 360 MPa (diameter = 10 mm). Pieces of indium foil (thickness = 0.3 mm, diameter = 9 mm) and lithium foil (thickness = 0.1 mm, diameter = 8 mm) were then placed on top of the bilayer pellets by pressing them all together under a pressure of 100 MPa. The pellets were pressed using two stainless steel rods, which were used as current collectors for both the positive electrode and the negative electrode. All the processes for preparing the electrochemical cells were performed in a dry Ar-filled glove box ([H_2_O] < 1 ppm).

## Author Contributions

A.S. synthesised the materials and wrote the paper. A.S., T.T., K.O., H.K. and H.S. characterised the synthesised materials. K.T. and Z.O. designed and supervised the project.

## Figures and Tables

**Table 1:** Simulated powder X-ray data for Li_2_TiS_3_ for a = 5.06 Å (The data were obtained using Cu-Kα radiation.)

*h*	*k*	*l*	2*θ*/deg	*d*/Å	Intensity
1	1	1	30.58	2.92	16
2	0	0	35.45	2.53	100
2	2	0	51.01	1.79	61
3	1	1	60.65	1.53	5
2	2	2	63.65	1.46	19
4	0	0	75.03	1.27	8
3	3	1	83.15	1.16	2
4	2	0	85.81	1.13	23
4	2	2	96.45	1.03	18

## References

[b1] Bruce P. G., Freunberger S. A., Hardwick L. J. & Tarascon J.-M. Li-O_2_ and Li-S batteries with high energy storage. Nature Mater. 11, 19–29 (2012). 22169914 10.1038/nmat3191

[b2] Delmas C., Brèthes S. & Ménétrier M. ω-Li*x*V_2_O_5_ - a new electrode material for rechargeable lithium batteries. J. Power Sources 34, 113–118 (1991).

[b3] Poizot P., Laruelle S., Grugeon S., Dupont L. & Tarascon J.-M. Nano-sized transition-metal oxides as negative-electrode materials for lithium-ion batteries. Nature 407, 496–499 (2000). 11028997 10.1038/35035045

[b4] Débart A., Dupont L., Patrice R. & Tarascon J.-M. Reactivity of transition metal (Co, Ni, Cu) sulphides versus lithium: The intriguing case of the copper sulphide. Solid State Sci. 8, 640–651 (2006).

[b5] Badway F., Cosandey F., Pereira N. & Amatucci G. G. Carbon metal fluoride nanocomposites high-capacity reversible metal fluoride conversion materials as rechargeable positive electrodes for Li batteries. J. Electrochem. Soc. 150(10), A1318–A1327 (2003).

[b6] Whittingham M. S. Role of ternary phases in cathode reactions. J. Electrochem. Soc. 123, 315–320 (1976).

[b7] Holleck G. L. & Drlscdll J. R. Transition-metal sulfides as cathodes for secondary lithium batteries. Electrochim. Acta 22, 647–655 (1977).

[b8] Whittingham M. S. Chemistry of intercalation compounds – metal guests in chalcogenide hosts. Prog. Solid State Chem. 12, 41–99 (1978).

[b9] Lindic M. H. *et al.* XPS investigations of TiO_y_S_z_ amorphous thin films used as positive electrode in lithium microbatteries. Solid State Ionics, 176, 1529–1537 (2005).

[b10] Hayashi A., Matsuyama T., Sakuda A. & Tatsumisago M. Amorphous titanium sulfide electrode for all-solid-state rechargeable lithium batteries with high capacity. Chem. Lett. 41, (9) 886–889 (2012).

[b11] Matsuyama T., Sakuda A., Hayashi A., Togawa Y., Mori S. & Tatsumisago M. Preparation of amorphous TiS_x_ thin film electrodes by the PLD method and their application to all-solid-state lithium secondary batteries. J. Mater. Sci. 47, 6601–6606 (2012).

[b12] Sakuda A. *et al.* Amorphous TiS_4_ positive electrode for lithium-sulfur secondary batteries. Electrochem. Commun. 31, 71–75 (2013).

[b13] Tarascon J. M., DiSalvo F. J., Eibschutz M., Murphy D. W. & Waszczak J. V. Preparation and chemical and physical-properties of the new layered phases Li_x_Ti_1-y_V_y_S_2_, Li_x_Ti_1-y_Cr_y_S_2_, Li_x_Ti_1-y_Fe_y_S_2_. Phys. Rev. B 28, 6397–6406 (1983).

[b14] Blandeau L., Ouvrard G., Calage Y., Brec R. & Rouxel Transition-metal dichalcogenides from disintercalation processes - crystal-structure determination and Mossbauer study of Li_2_FeS_2_ and its disintercalates Li_x_FeS_2_ (0.2 ≤ x ≤ 2). J. J. Phys. C 20, 4271–4281 (1987).

[b15] Takada K. *et al.* Electrochemical reduction of Li_2_FeS_2_ in solid electrolyte. J. Electrochem. Soc., 148, A1085–A1090 (2001).

[b16] Kraus W. & Nolze G. POWDER CELL - A program for the representation and manipulation of crystal structures and calculation of the resulting X-ray powder patterns. J. Appl. Crystallogr. 29, 301(1996).

[b17] Izumi F. & Ikeda T. A Rietveld-analysis program RIETAN-98 and its applications to zeolites. Mater. Sci. Forum, 321–324, 198–203 (2000).

[b18] Ujiie S., Hayashi A. & Tatsumisago M. Structure, ionic conductivity and electrochemical stability of Li_2_S-P_2_S_5_-LiI glass and glass-ceramic electrolytes. Solid State Ionics, 211, 42–45 (2012).

[b19] Ohtomo T., Hayashi A., Tatsumisago M. & Kawamoto K. Glass electrolytes with high ion conductivity and high chemical stability in the system LiI-Li_2_O-Li_2_S-P_2_S_5_. Electrochem. 81, 428–431 (2013).

